# Biotransformation of Flavonoids Improves Antimicrobial and Anti-Breast Cancer Activities In Vitro

**DOI:** 10.3390/foods10102367

**Published:** 2021-10-05

**Authors:** Yanpeng Hao, Zuchen Wei, Zhi Wang, Guiying Li, Yang Yao, Baoqing Dun

**Affiliations:** 1 Institute of Crop Science, Chinese Academy of Agricultural Sciences, No. 80 South Xueyuan Road, Haidian District, Beijing 100081, China; haoyanpeng@caas.cn (Y.H.); weizuchen@163.com (Z.W.); wangzhi@caas.cn (Z.W.); liguiying@caas.cn (G.L.); yaoyang@caas.cn (Y.Y.); 2 Laboratory for Green Cultivation and Deep Processing of Three Gorges Reservoir Area’s Medicinal Herbs, College of Life Science & Engineering, The Chongqing Engineering, Chongqing Three Gorges University, No. 666 Tianxing Road, Wanzhou District, Chongqing 404000, China

**Keywords:** flavonoids, biotransformation, methylation, antimicrobial, anti-breast cancer

## Abstract

Coarse cereals are rich in flavonoids, which are bioactive substances with a wide range of functions. Biotransformation is considered an emerging approach to methylate flavonoids, displaying prominent regio- and stereoselectivity. In the current study, liquiritigenin, naringenin, and hesperidin flavonoids were biotransformed using *O*-methyltransferases that were heterologously expressed in *Saccharomyces cerevisiae* BJ5464-NpgA. Nuclear magnetic resonance (NMR) spectroscopy was used together with high-resolution mass spectroscopy analysis to determine the structures of the resulting methylated transformants, and their antimicrobial and antiproliferation activities were also characterized. Among the five methylated flavonoids obtained, 7-methoxy-liquiritigenin had the strongest inhibitory effect on *Candida albicans* SC5314 (*C. albicans* SC5314), *Staphylococcus aureus* ATCC6538 (*S. aureus* ATCC6538), and *Escherichia coli* ATCC25922 (*E. coli* ATCC25922), which increased 7.65-, 1.49-, and 0.54-fold in comparison to the values of their unmethylated counterparts at 200, 250, and 400 μM, respectively. The results suggest that 3′-methoxyhesperetin showed the best antiproliferative activity against MCF-7 cells with IC_50_ values of 10.45 ± 0.45 µM, which was an increase of more than 14.35-fold compared to that of hesperetin. These results indicate that methylation enhances the antimicrobial activities and antiproliferative effects of flavonoids. The current study provides an experimental basis for further research on flavonoids as well as flavonoid-containing crops in the development of antimicrobial and anti-breast cancer drugs in addition to supplementary and health foods. The biotransformation method is ideal, as it represents a means for the sustainable production of bioactive flavonoids.

## 1. Introduction

Flavonoids are a type of plant secondary metabolite that are widely present in many minor grains, such as Tartary buckwheat, quinoa, and barley [1,2,3]. In addition to being diverse in variety, flavonoids also possess complex structure types, and they perform many bioactive functions, including antioxidation effects [4,5], antiproliferative effects [6], and antibacterial effects [7,8]. Flavonoids have been commonly used in bacteriostatic therapy and are considered to possess the potential to replace some antibiotics; quercetin, for example, similarly to existing antibiotics, has the potential to treat *Pseudomonas aeruginosa* infection and its use as an alternative may help to ease the selection pressure due to antibiotic overuse [9]. However, many natural flavonoids present many disadvantages, such as low content, poor water solubility, and unstable bioavailability. For example, adverse reactions caused by puerarin have been reported in its clinical application, including fever, allergies, and hemolysis [10], which are related to its poor solubility and low bioavailability. Therefore, the use of current technology has been applied to address these problems through the development of derivatives via structural modification.

The main methods of structural modification include the introduction of halogen or other active groups. Common active groups are methyl, acyl, and glycosyl. Acylation modification can affect the ability of flavonoid to attack free radicals and regulate its interaction with proteins, enzymes, and specific cells [11], subsequently improving their antioxidation [12,13], anti-inflammatory, and other lipid-lowering [14] abilities. The introduction of different active groups into flavonoids can change the conformation of flavonoids, enhance their biological activity, and even produce new physiological functions. Methylquercetin metabolized in filamentous fungi showed lower cytotoxicity on leukemic HL-60 cells than quercetin [15]. Moreover, in an in vivo study on drug-resistant seizures, 4′,7-dimethyl ether naringenin performed better than naringenin [16].

At present, chemical catalysis and biocatalysis are emerging methods used to modify flavonoid structure. Biocatalysis methods mainly use enzymes secreted by microorganisms, while chemical catalysis is carried out via specific chemical reactions. Biocatalysis is considered to be green and eco-friendly technology, which has sustainable development prospects [17]. Since 2000, a variety of microorganisms have exhibited abilities to convert flavonoids, and some microorganisms were also found to naturally and directly synthesize flavonoids as a result of their own endogenous metabolism [18]. The biocatalytic production of flavonoids offers many advantages, such as fast production, low environmental pollution, and large-scale production. However, there is a scarcity of research on flavonoid biocatalysis and the bioactivity of the resulting product.

Therefore, the aim of the current work was to determine whether biocatalytic methods can be used to achieve methylation modification of flavonoids and to characterize the potential improvement in the biological activity, structural stability, and bioavailability of these methylated products. This study contributes to realizing the green, economic, fast, efficient, and sustainable development of the production of bioactive flavonoids.

## 2. Materials and Methods

### 2.1. Chemicals and Regents

Primary samples (liquiritigenin, naringenin, and hesperetin) and other compounds were all obtained from Shanghai Yuanye Bio-Technology Co., Ltd. (Shanghai, China). All media components were provided by Clontech Laboratories, Inc. (Los Angeles, CA, USA). Methylene blue, Luria–Bertani medium, and potato dextrose broth medium were purchased from BD-Pharmingen (San Diego, CA, USA). Other reagents were of analytical grade.

### 2.2. Biotransformation Procedures

Biotransformation procedures were performed in a laboratory according to the methods of Wang et al. [19]. Briefly, the *Saccharomyces cerevisiae* BJ5464-NpgA strain was used for to host the plasmids YEpADH2-LEU-HsOMT and YEpADH2-LEU-LtOMT for expression of both O-methyltransferases HsOMT and O-methyltransferases LtOMT, respectively. These expression constructs had previously been successfully prepared by our laboratory [19]. In the biotransformation procedure, the OMT vector was transformed into *Saccharomyces cerevisiae* BJ5464-NpgA host, and dropout minimal medium agar plates were used to select transformants on SC-Leu (yeast synthetic dropout medium without leucine). Recombinant yeast cells were cultured in 100 mL medium (containing 25 mL of the appropriate SC-Leu minimal dropout medium) to an OD600 of 0.6. The medium was then mixed with an equal volume of YPD medium (1% yeast extract, 2% peptone, and 1% dextrose). Experimental flavonoids dissolved in dimethyl sulfoxide (DMSO) were also added to the cultures (10 μg/mL, final concentration), where appropriate, when the YPD medium was supplied. The fermentation temperature was maintained at 30 °C with shaking at 220 rpm for an additional 48 h until the wet cell weight reached 1.28 ± 0.18 g. The biotransformation efficiency was determined based on HPLC.

### 2.3. Extraction and Purification of the Products

An equal volume of ethyl acetate was used to extract the fermentation cultures three times. Crude extract was first purified by silica gel column chromatography to obtain seven fractions (methanol/water 100:0, 90:10, 80:20, 70:30 60:40, 50/50, and 10/90, *v*/*v*), and subsequent extracts were dried in vacuo. Quantitative HPLC analysis was conducted on an AB SCIEX HPLC instrument equipped with a C_18_ column (Cosmosil YMC-Pack ODS-AM, 12, 20, and 250 mm) to purify and obtain the target compound.

### 2.4. Chemical Identification

Samples were routinely analyzed using an AB SCIEX 4000 HPLC instrument (Waltham, MA, U.S.) applied onto an Agilent Eclipse Plus C_18_ RRHD column (1.8 µm, 2.1 mm × 50 mm). Samples were first eluted using acetonitrile–water (10–95%) for 20 min and then 95% (acetonitrile–water) for 10 min, 10% for 5 min, and finally 10% (acetonitrile–water) for 5 min with 0.5 mL/min.

### 2.5. Structural Characterization of the Target Compound by LC-MS

Products were first dried in vacuo and then restructured in methanol. HPLC–HRESI -MS and MS–MS spectra were acquired on an AB SCIEX HPLC coupled with an Agilent QTOF 6530 instrument (capillary: 3.6 kV, cone voltages: 40–150 V). The collision energy was 35 V and was calibrated each time with a standard calibration solution (Agilent) (*m*/*z* 150–800).

### 2.6. Structural Characterization of the Target Compound by NMR

^1^H NMR, ^13^C NMR, HSQC, and HMBC (2D NMR) spectra were obtained using an Agilent 600 DD2 spectrometer. Per million (ppm) and Hz (J values) were considered chemical shift values (δ) and the coupling constants. The residual solvent peak of DMSO-*d*6 were used as references for chemical shifts.

### 2.7. Antimicrobial Biological Activity Assay

Antimicrobial biological activities of the tested compounds were evaluated as Zhong et al., with some modifications [20]. Gram-positive and -negative bacteria and fungi (*S. aureus* ATCC6538, *E. coli* ATCC25922, and *C. albicans* SC5314) were selected for evaluation in the present study. *S. aureus* ATCC6538 and *E. coli* ATCC25922 were cultured with Luria-Bertani medium, while for *C. albicans* SC5314, potato dextrose broth medium was used for 24 h at 37 °C (bacterial suspension in 1 × 106 CFU/mL). The experimental concentration ranges were from 75 to 400 µM. Each strain was cultured until an OD value of 0.6–0.8 was reached and then diluted 1000×. Each strain was, respectively, transferred into a 96-well plate at 100 µL/well (90 µL bacterial suspension and 10 µL sample solution) after adding different concentrations of samples, followed by incubation for 12–16 h. The optical density at 650 nm (OD_650_) of *C. albicans* SC5314 was measured after TTC staining, while *S. aureus* ATCC6538 and *E. coli* ATCC25922 were stained for CCK8 before measurement. The minimal bactericidal concentration (MBC) and minimal inhibitory concentration (MIC) were simultaneously determined at a concentration of 15–400 µM.

### 2.8. Antiproliferative and Cytotoxicity Activities Assay

Analysis of the antiproliferative activity of the test compounds was performed according to Zhu et al. [21], with some modifications. MCF-7 human breast cancer cells were provided by the Cell Resource Center of the Chinese Academy of Sciences (Shanghai, China). Cell proliferation and cytotoxicity of anticancer activity were assessed using the methylene blue assay. For cell proliferation and cytotoxicity, MCF-7 cells were culture in a 96-well plate at 1.5 × 10^5^ cells/well and at 3 × 10^5^ cells/well, respectively. Various concentrations (75–400 µM) of the test samples were added to a growth medium containing MCF-7 cells followed by cultivation for 16 h. Following incubation for 72 h, cell proliferation was measured with an absorbance of 570 nm using a microplate reader (Bio-Rad, Hercules, MA, USA), while cell cytotoxicity measurement was carried out 24 h after incubation.

### 2.9. Data Analysis

Data in present study were processed with one way ANOVA and Tukey’s test by SPSS (Statistics for Social Science) version 17.0 (IBM, New York, NY, USA). The figure was conducted in GraphPad Prism 8.0.29 (GraphPad Software, San Diego, California, CA, USA). All experiments were conducted three times. Data were imported with mean ± SD, and the significant differences were considered as *p* < 0.05.

## 3. Results

### 3.1. Biotransformation

**Figure 1:**
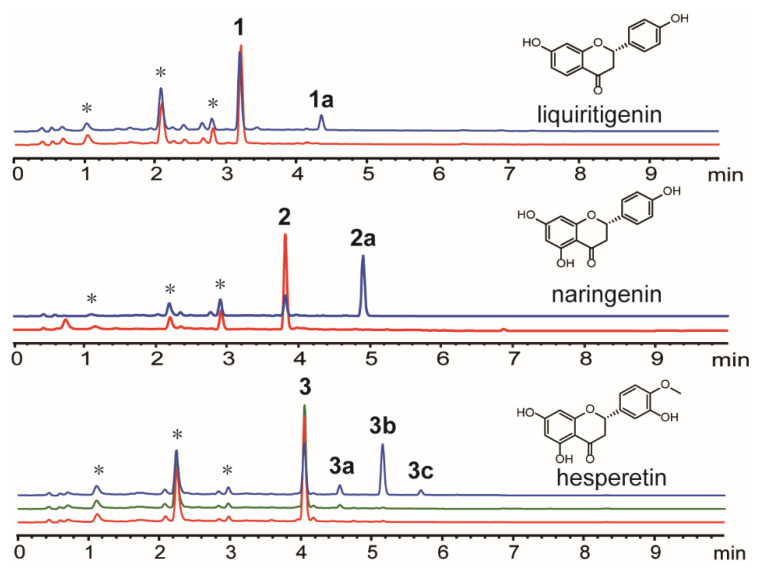
Biotransformation of flavonoid substrates by HsOMT and LtOMT. * represents equivalent endogenous substrate.

The two enzymes used for biotransformation, HsOMT and LtOMT, exhibited different catalytic abilities (Figure 1 and Table 1).

Liquiritigenin, naringenin, and hesperetin were catalyzed by HsOMT in *Saccharomyces cerevisiae* BJ5464-NpgA to obtain at least one product. Liquiritigenin was catalyzed by HsOMT in *Saccharomyces cerevisiae* BJ5464-NpgA to obtain one product, 1a, whose biotransformation yield was 15.64%. Similarly, naringenin was catalyzed by HsOMT in *Saccharomyces cerevisiae* BJ5464-NpgA to obtain one product, 2a, whose biotransformation yield was 73.12%. Hesperetin had three methylated products, two monomethylated isomers (3a and 3b), and one double methylated product (3c). Hesperetin was catalyzed by HsOMT and LtOMT to 7-methoxyhesperetin (Table 1).

The biotransformation yield of hesperetin was 51.34%. NMR was measured to further characterize the structure. Liquiritigenin and naringenin were catalyzed by LtOMT but failed to obtain products explaining the regioselectivity of LtOMT, in which LtOMT is specific to the phenolic hydroxyl residing at the ortho position and the aromatic carbon bearing the carbonyl.

### 3.2. Structure Characterization

**Figure 2:**
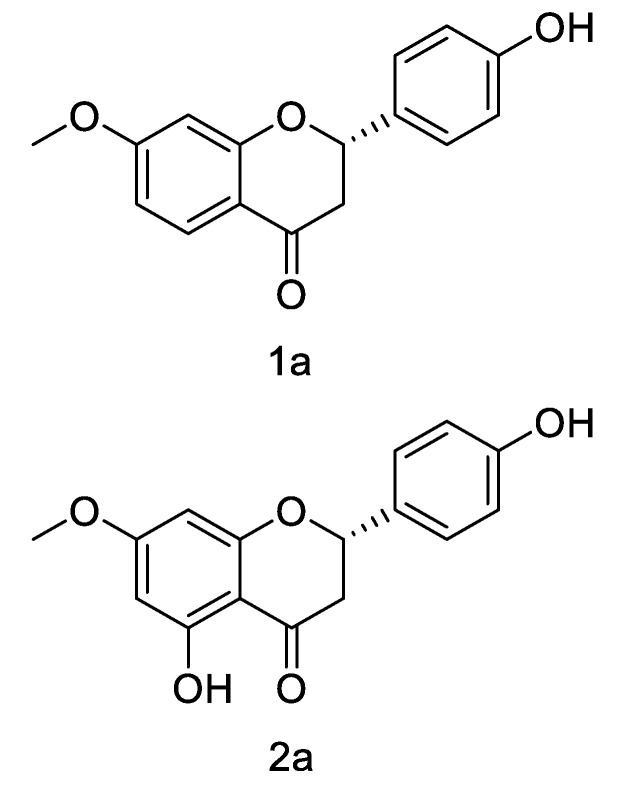

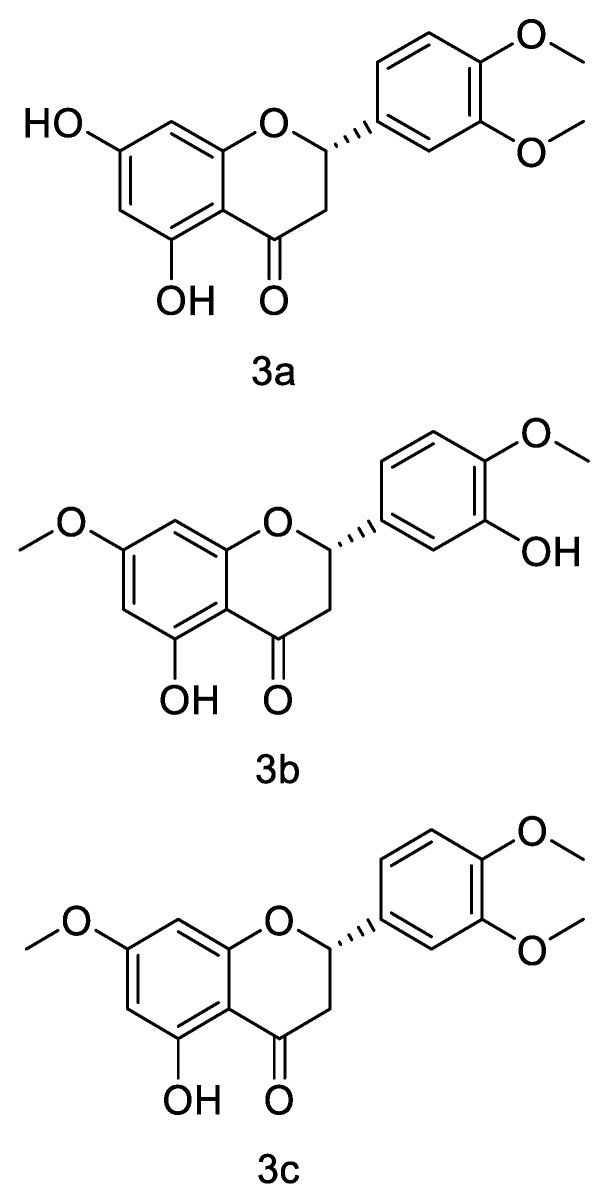
Structures of compounds 1a, 2a, 3a, 3b, and 3c, as identified by NMR.

The structures of 1a, 2a, 3a, 3b, and 3c were elucidated by analyzing the NMR spectroscopic data of the purified compounds. Analysis of 1D and 2D NMR data (Figure 2, Table 2 and Table 3) showed considerable similarities between the structures of 1a and glycyrrhizin, except for the absence of a methoxy group. In HMBC, correlation between 7-OMe (*δ*_H_ 3.80) and aromatic carbon at *δ*_C_ 165.67 suggests that the methoxy group was located at C-7. Compound 1a exhibited similar NMR spectra (Table 2) to those obtained for 7-methoxyglycyrrhizin. Therefore, the structure of 1a was assigned as 7-methoxyglycyrrhizin. In the same way, by comparing ^1^H NMR and ^13^C NMR of 2a with the corresponding substrate, naringenin, the presence of an additional methoxy group was observed. The positions of the methoxy groups of compounds 2a were determined at C-7 by HMBC correlation between 7-OMe (*δ*_H_ 3.78) and aromatic carbon at *δ*_C_ 167.45 in 2a. The structure of 2a was finally elucidated as 7-methoxynaringenin. The positions of the methylation of compounds 3a, 3b, and 3c were located at C-3*′*, C-7, and both C-3*′* and C-7, respectively, by comparing 1D and 2D NMR data for 3a, 3b, and 3c with those for their common substrate, hesperetin. Therefore, the structures of 3a, 3b, and 3c were determined to be 3*′*-methoxyhesperetin, 7-methoxy-hesperetin, and 3*′*, 7-dimethoxyhesperetin, respectively.

### 3.3. Antimicrobial Biological Activity

**Figure 3:**
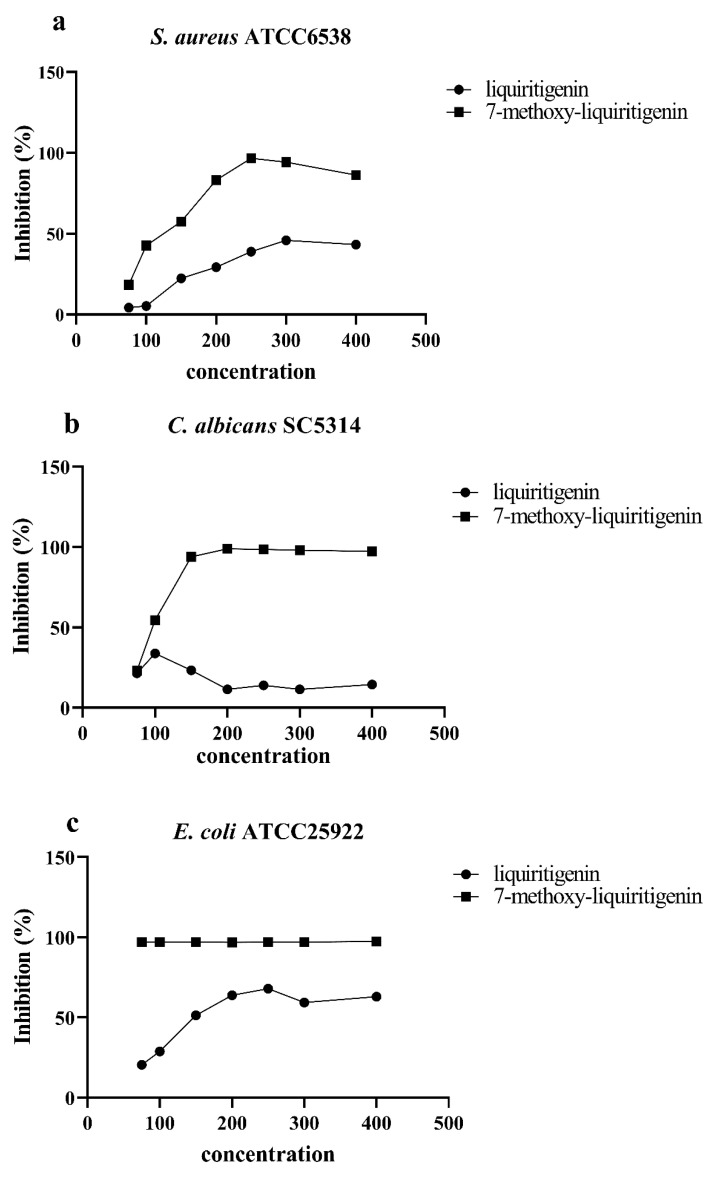

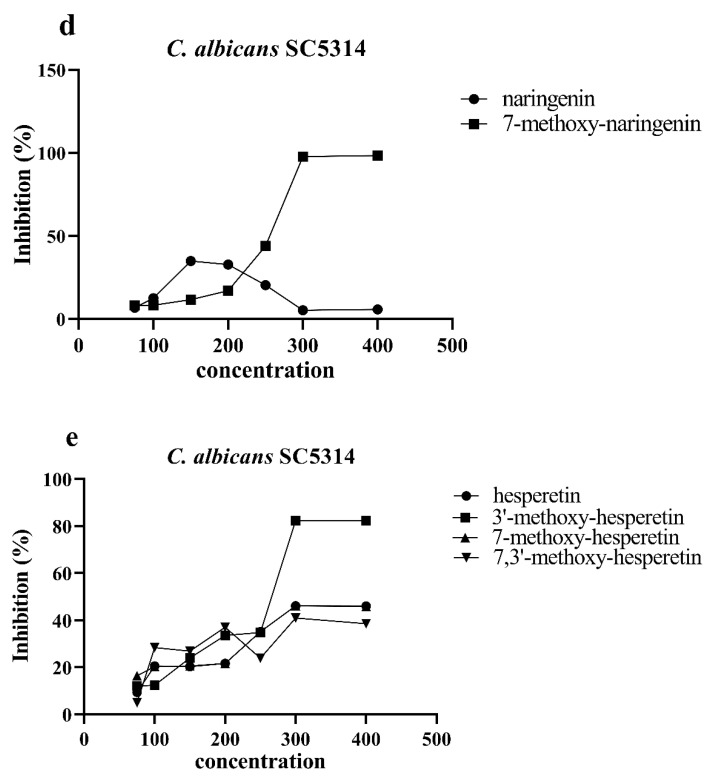
Inhibitory effect of (**a**,**b**) liquiritigenin and its methylated product 1a on *S aureus* ATCC6538, *C. albicans* SC5314, and *E. coli* ATCC25922; (**c**,**d**) naringenin 3 and its methylated product 2a on *C. albicans* SC5314; and (**e**) hesperidin and its methylated products 3a, 3b, and 3c on *C. albicans* SC5314. Data are presented as mean ± SD.

The results of the antimicrobial biological activity assays of the samples are shown in Figure 3a–e. Three microbes (C*. albicans* SC5314, *S*. *aureus* ATCC6538, and *E. coli* ATCC25922) were considered in the assay for antimicrobial activity. Sample 1a effectively inhibited the growth of the three microbes. The strongest antimicrobial activity against *S. aureus* ATCC6538, *C. albicans* SC5314, and *E. coli* ATCC25922 was 1a at the dosage of 250, 200, and 400 μM, which increased by 148.77, 765.64, and 54.41% compared to liquiritigenin, respectively (Figure 2(1a–3c)). There was significant difference in the inhibition of *C. albicans* SC5314 and *E. coli* ATCC25922 by liquiritigenin, compared with 7-methoxy-liquiritigenin at the concentration of 75–400 µM (*p* < 0.05). In particular, the antimicrobial l activity against *E. coli* ATCC25922 remained above 95% at the experimental concentration. However, naringenin and hesperetin were only observed to possess antimicrobial activity against *C. albicans* SC5314 (Figure 3d,e). From 75 to 200 μM, the antimicrobial activities of naringenin were higher than that of 2a, while a reverse trend was observed when the concentration increased over 200 μM (Figure 3d). The highest antimicrobial activity of 2a increased by 182.12% compared to that of naringenin (Figure 3d). The variations in the in vitro antimicrobial activities of hesperetin and its products (3a, 3b, and 3c) are displayed in Figure 3e, which shows that 3a performed the best. The antimicrobial activities of sample 3a and 3b increased with the increase in concentration, while 3c performed the best at 300 μM (Figure 3e). Meanwhile, compared with that of the other products, the antimicrobial activity of 3c sharply increased at concentrations higher than 250 μM (*p* < 0.05). The MIC and MBC of products were shown in Table 4.

### 3.4. Antiproliferation Activity

**Figure 4:**
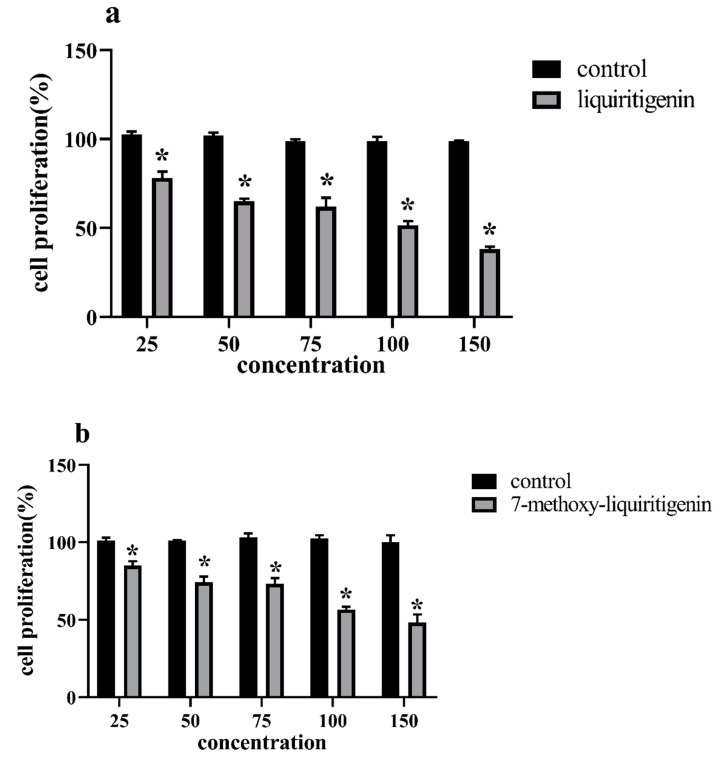

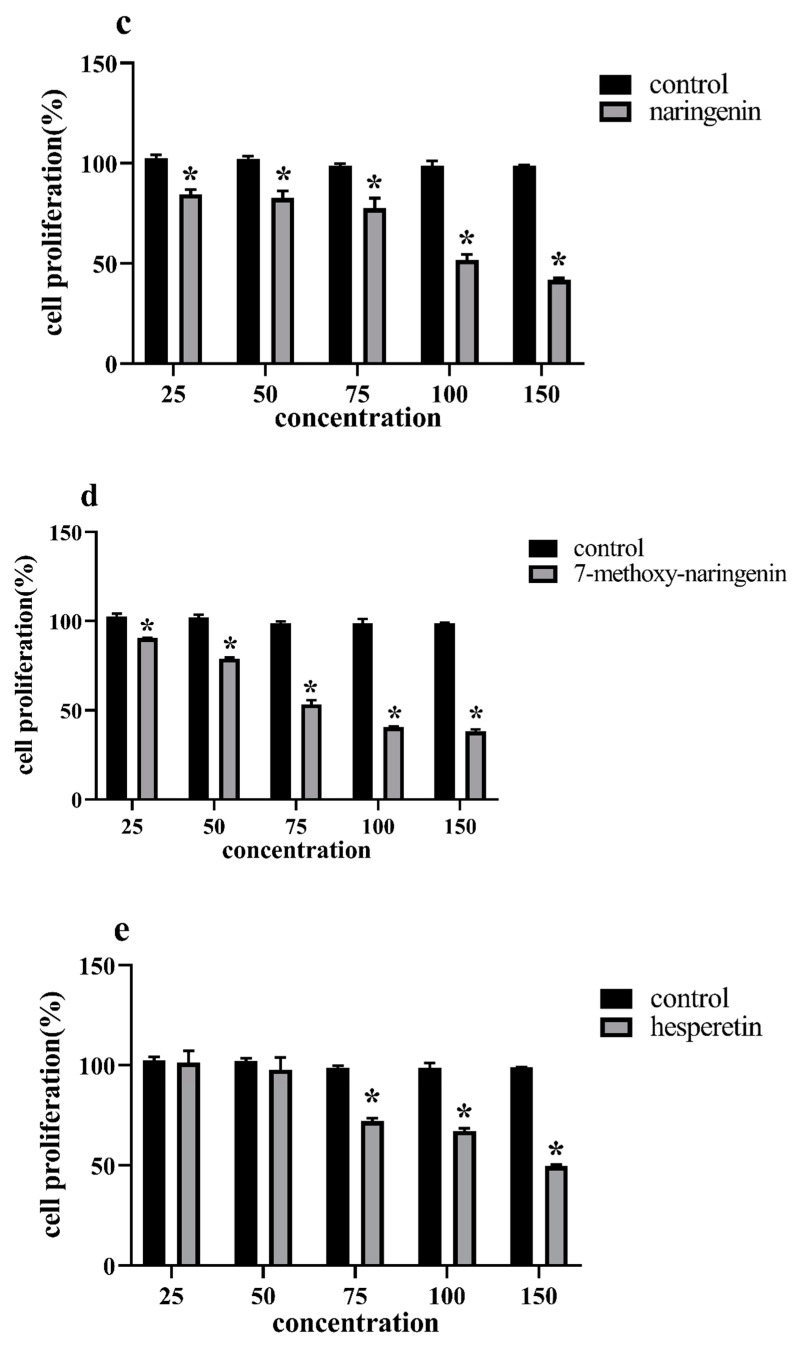

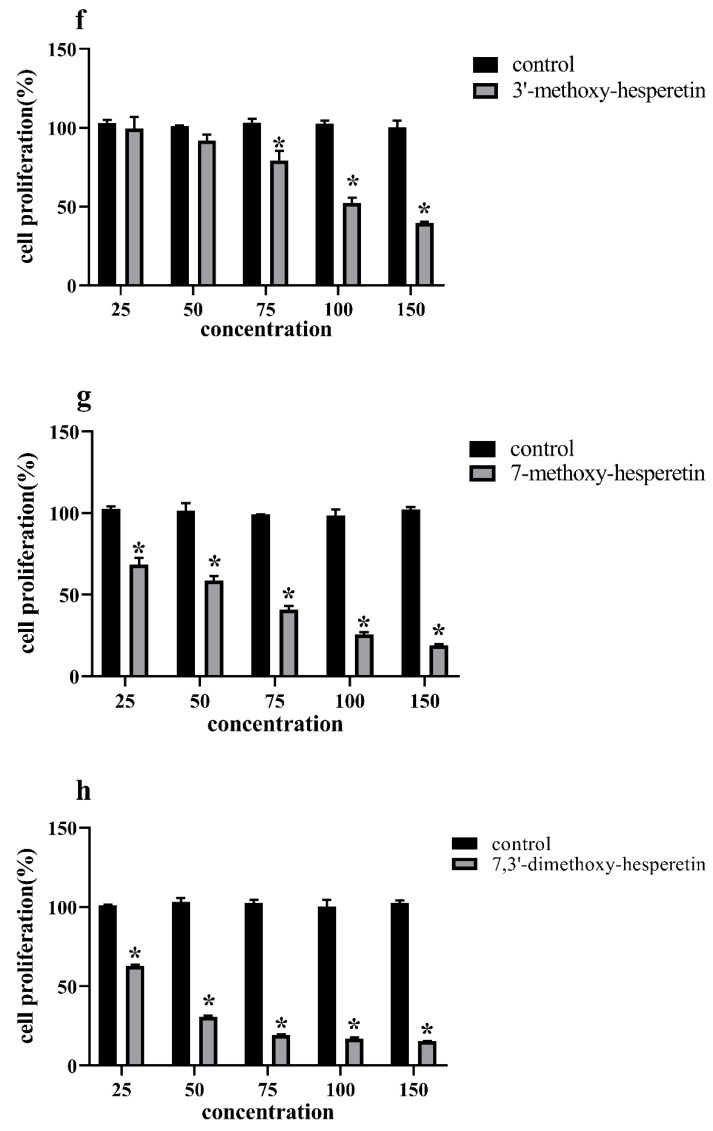
Inhibition of proliferation of MCF-7 human breast cancer cells by (**a**,**b**) liquiritigenin and 7-methoxy-liquiritigenin; (**c**,**d**) naringenin and 7-methoxynaringenin; and (**e**–**h**) hesperetin, 3′-methoxyhesperetin, 7-methoxyhesperetin and 7,3′-dimethoxyhesperetin. (mean ± SD, n = 3). * Indicates a significant difference compared to the control (*p* < 0.05).

MCF-7 cell proliferation was inhibited in a dose-dependent manner after exposure to liquiritigenin, naringenin, and hesperetin (Figure 4a–h, Table 5).

Cell proliferation was inhibited after exposure to liquiritigenin and 1a in a dose-dependent manner (Figure 4a,b) (IC _50_ of 100.94 ± 1.83 μM, 11.23 ± 0.60 μM) (Table 5). Compared to liquiritigenin, the antiproliferation potency of compound 1a increased 8.99-fold. Naringenin 2 and 2a showed good antiproliferative activity compared to that of the control culture (Figure 4c,d) (*p* < 0.05). Cell proliferation was inhibited after exposure to naringenin and 2a (IC _50_ of 125.53 ± 2.76 μM, 93.64 ± 1.06 μM). The antiproliferation potency of compound 2a was enhanced by 1.34-fold compared to that of naringenin. Methylation products of hesperidin (3a, 3b, and 3c) resulted in products that showed comparable antiproliferative activities compared to the control (*p* < 0.05). Cell proliferation was inhibited after exposure to 3a, 3b, and 3c, and their IC _50_ values were 10.45 ± 0.45 μM, 30.74 ± 0.72 μM, and 31.00 ± 1.44 μM, respectively (Figure 4e–h, Table 5). The IC _50_ was over 150 μM for MCF-7 cells compared to the control (*p* < 0.05) (Table 5). Compound 3a showed 2.01- and 1.97-fold increases in antiproliferation potency compared to that of 3b and 3c, respectively.

### 3.5. Cytotoxicity Studies

**Figure 5:**
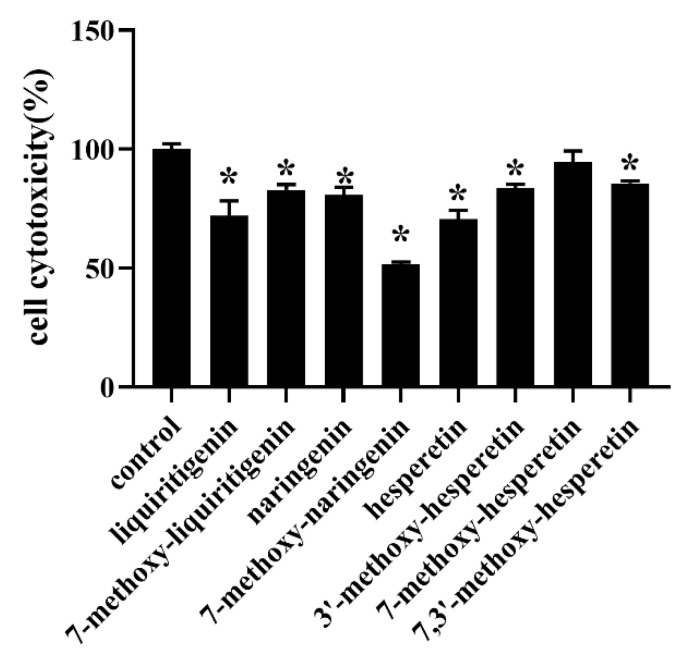
Cytotoxicity of liquiritigenin, 7-methoxy-liquiritigenin, naringenin, 7-methoxynaringenin, hesperetin, 3′-methoxyhesperetin, 7-methoxyhesperetin, and 7,3′-dimethoxyhesperetin on MCF-7 breast cancer cells (mean ± SD, n = 3). Data are presented as mean ± SD. * Indicates a significant difference compared to the control (*p* < 0.05).

The cytotoxicity of liquiritigenin, 1a, naringenin, 2s, hesperetin, 3a, 3b, and 3c at a concentration corresponding to the IC _50_ on MCF-7 cells is shown in Figure 5. The cytotoxicity of 150 μM hesperetin on MCF-7 cells is shown in Figure 4. No cytotoxicity was observed for 7-methoxyhesperetin on MCF-7 cells. Slight cytotoxicity was observed for liquiritigenin, naringenin, hesperetin, 7-methoxynaringenin, 7-methoxy-liquiritigenin, 3′-methoxyhesperetin, and 7, 3′-dimethoxyhesperetin on MCF-7 cells.

## 4. Discussion

Flavonoids and their derivatives can potentially be used in clinical treatment due to their notable antimicrobial, anticancer, and anti-inflammatory effects [22]. Therefore, scientists are continuously developing technology such as for structural modification of flavonoids to alter their biological activities [11,12,13,14]. In the present study, LtOMT and HsOMT, as two orthologous O-methyltransferases (OMTs) in two fungal benzenediol lactones, were considered for carrying out biotransformation [19]. The enzymes HsOMT and LtOMT exhibited variable catalytic abilities in the modification of three substrates (liquiritigenin, naringenin, and hesperetin). NMR analysis found that, in 1a and 2a, the methyl group was added by HsOMT at the seventh position of liquiritigenin and naringenin, respectively, replacing the hydrogen in the hydroxyl group and becoming a methoxy group. However, three products were obtained in the reaction with hesperetin: 3a (catalyzed by HsOMT); 3b (catalyzed by LtOMT); and 3c (catalyzed by LtOMT and HsOMT).

The bioactivity was significantly influenced by methylation [23], but not all the transformed products possessed the desired properties in the present study. Previous studies reported that pure naringenin showed poor antimicrobial activities, and free hesperetin also did not demonstrate activity against either *E. coli* or *S. aureus* [24,25]. Similarity, naringenin, hesperetin, and its methylation products (2a, 3a, 3b, and 3c) also failed to inhibit the growth of *S. aureus* ATCC 6538 and *E. coli* ATCC25922 in the present study. The effect of antimicrobial activities was particularly relevant to the conversion of highly hydrophilic groups and steric hindrance of reactive sites [26]. Magozwi et al. revealed that methylation of the hydroxyl groups on C-3 or C-7 reduced the antimicrobial activity of flavonoids [27]. Research on quercetin indicated that the loss of a hydroxyl group at C-3 was associated with low activity [27]. Here, the results for the methylation product of liquiritigenin on *S. aureus* ATCC6538, *E. coli* ATCC25922, and *C. albicans* SC5314 indicated that methylation did not lower the antimicrobial activity compared with liquiritigenin. In previous studies, liquiritigenin demonstrated a strong antibacterial effect on *E. coli* [28].

The anticancer ability of flavonoids has been the subject of great research interest in recent years. Previous studies focusing on anticancer abilities have demonstrated that MCF-7 breast cancer cell proliferation was inhibited in a dose-dependent manner after exposure to liquiritigenin, naringenin, and hesperetin [29,30,31]. Liquiritigenin was found to effectively inhibit the invasiveness of breast cancer cells by inhibiting DNA methyltransferase (DNMT) activity and increasing (breast cancer 1) BRCA1 expression [29], and naringenin treatment regulated endoplasmic reticulum (ER)-stress mediation to prevent breast cancer cell proliferation [32]. Meanwhile, hesperetin decreased the expression of aromatase and inhibited estrogen production in MCF-7 breast cancer cells [33]. The anticancer ability of flavonoids was influenced by the range of O-methylation, while the ability of methylated quercetin to inhibit HL60 leukemia cell proliferation was weaker than that of its unmethylated counterpart, and poly-O-methylated nobiletin and tangeretin showed the reverse trend in human squamous cell carcinoma [34]. Therefore, the anticancer performance of methylated flavonoids was variable and associated with the position of methylation and the type of cancer cell. Compounds 1a, 2a, 3a, 3b, and 3c also showed good performance in terms of anticancer activities. Katayama et al. reported that the methylation in 4-C of the B-ring was associated with promising potential in treating cancer [35]. However, 3′-O-methylation was also found to enhance the antiproliferative function of nobiletin, and the highly methylated flavonoid 3′,4′,7-trimethoxyflavone showed better inhibitory activity against the breast cancer resistance protein compared to the minimally methylated acacetin. In the present study, 3c displayed the best antiproliferation ability [35,36]. Interestingly, methylation products also showed good inhibition ability against MCF-7 breast cancer cells. The substrates in our study, for which a methyl group was added, appear to regulate some important functions in cell, such as gene regulation, epigenetics, and cellular energy status [34]. In addition to the antiproliferation ability of the methylation products, it is also worth discussing their toxicity. It has been reported that the existence of a 4-carbonyl group and 2–3 double bond is associated with cytotoxicity [37,38]. The cytotoxicity of the samples was demonstrated at the concentration of IC _50_. It can be clearly observed that 3b and 2a showed the lowest and highest cytotoxicity among the samples, respectively. Naringenin had lower cytotoxicity than that of liquiritigenin, which was in accordance with the results of previous reports that the number of hydroxyl groups was negatively associated with cytotoxicity [39]. In fact, highly methoxylated products demonstrate higher toxicity than those that are less methoxylated, but the increase in hydroxyl groups decreased the hydrophobicity, thereby lowering product toxicity [39]. Therefore, products of hesperetin ultimately showed better performance in terms of cytotoxicity.

## 5. Conclusions

The current study confirmed that three of the examined flavonoids tended to be transformed by HsOMT and not LtOMT. Among these compounds, naringenin was transformed by HsOMT with the highest conversion yield of 78%. As a result, five known flavonoids were extracted and isolated from fermented cultures, and their structures were subsequently characterized by NMR. The results indicate that HsOMT easily methylates the free aromatic hydroxyl groups on ring A of flavonoids and could be developed as a regioselective biocatalyst for the methylation of phenolic natural products. The results of the antimicrobial experiment show that 7-methoxy-lliquiritigenin had the strongest inhibitory effect on *C. albicans* SC5314 and *S. aureus* ATCC6538, with MIC values of 25 and 75 µM, respectively, which were increased 3- and 1.25-fold compared to the unmethylated substrate. Additionally, 3’-methylhesperidin and 7-methylhesperidin had stronger inhibitory effects on *C. albicans* SC5314 than those of hesperidin and 7,3’-dimethylhesperidin. This may be due to the steric hindrance caused by the methylation of two hydroxyl groups of 7,3’-dimethylhesperetin, which affected the antimicrobial activity. From the experiment of antiproliferation and cell cytotoxicity assays on MCF-7 cells, it can be concluded that 7-methoxy-liquiritigenin had increased antiproliferative and cytotoxicity activities compared with liquiritigenin, and similar results were obtained for 7-methylnaringenin and naringenin. The results demonstrate that, compared with hesperetin, the derivatives 7-methylhesperetin, 3’-methylhesperetin, and 7,3’-dimethylhesperetin had an increased antiproliferation effect but decreased cytotoxicity, and that 7-methylhesperetin had no cytotoxicity. These results indicate that methylation can enhance the antimicrobial activity and antiproliferative effect of flavonoids. The current study provides an experimental basis for the further research on flavonoids as well as flavonoid-containing crops in the development of antimicrobial and anti-breast cancer drugs in addition to supplementary and health foods. Biotransformation is an ideal method, as it represents a means for the sustainable production of flavonoids.

## Figures and Tables

**Table 1:** Biotransformation products and yields of flavonoid substrates by Hs-OMT and Lt-OMT.

Number	Name	Biocatalytic Enzymes	Molecular Formula	Biotransformation Yield (%)
1a	7-methoxy-liquiritigenin	Hs-OMT	C_16_H_14_O_4_	15.64 ± 1.61
2a	7-methoxy-naringenin	Hs-OMT	C_16_H_14_O_5_	73.12 ± 3.47
3a	3’-methoxy-hesperetin	Hs-OMT	C_17_H_16_O_6_	8.42 ± 1.33
3b	7-methoxy- hesperetin	Lt-OMT	C_17_H_16_O_6_	3.73 ± 0.84
3b	7-methoxy-hesperetin	Hs-OMT	C_17_H_16_O_6_	43.81 ± 0.02
3c	7,3’-dimethoxy-hesperetin	Hs-OMT	C_18_H_18_O_6_	3.80 ± 0.84

**Table 2:** ^1^H (600 MHz) and ^13^C NMR (150 MHz) data of 1a and 2a in DMSO-*d*_6_.

No.	1a		2a	
	*δ*_C_	*δ*_H_ (Multi, *J* in Hz)	*δ*_C_	*δ*_H_ (Multi, *J* in Hz)
2	79.26	5.48, brd (13.4)	78.65	5.48, brd (14.8)
3*α*	43.14	3.15, m	42.05	3.30, dd (17.1, 14.8)
3*β*		2.66, m		2.72, dd (17.1, 2.5)
4	190.42		197.00	
5	128.03	7.71, d (8.6)	163.22	
6	109.87	6.64, d (8.6)	94.67	6.07, d (1.7)
7	165.67		167.45	
8	101.01	6.58, s	93.81	6.10, brs
9	163.30		162.90	
10	114.46		102.62	
1*′*	129.16		128.70	
2*′*	128.32	7.33, d (7.3)	115.19	7.32, d (8.2)
3*′*	115.17	6.78, d (7.3)	128.40	6.79, d (8.2)
4*′*	157.73		157.79	
5*′*	115.17	6.78, d (7.3)	128.40	6.79, d (8.2)
6*′*	128.32	7.32, d (7.3)	115.19	7.32, d (8.2)
7-OMe	55.81	3.80, s	55.92	3.78, s

**Table 3:** ^1^H (600 MHz) and ^13^C NMR (150 MHz) data for 3a, 3b, and 3c in DMSO-*d*_6_.

No.	3a		3b		3c	
	*δ*_C_	*δ*_H_ (Multi, *J* in Hz)	*δ*_C_	*δ*_H_ (Multi, *J* in Hz)	*δ*_C_	*δ*_H_ (Multi, *J* in Hz)
2	78.48	5.48, dd (12.5, 2.4)	78.41	5.48, d (11.4)	78.70	5.53, dd (12.6, 2.1)
3*α*	42.10	3.32, m	42.13	3.26, dd (17.2, 11.4)	42.17	3.38, m
3*β*		2.71, dd (17.1, 2.4)		2.75, d (17.2)		2.75, dd (17.0, 2.1)
4	196.22		196.79		196.86	
5	163.46		163.18		163.20	
6	95.85	5.89, brs	94.63	6.08, brs	94.71	6.09, brs
7	166.72		167.42		167.46	
8	95.03	5.91, brs	93.82	6.11, brs	93.86	6.13, brs
9	162.82		162.73		162.79	
10	101.73		102.63		102.61	
1*′*	130.97		130.97		130.81	
2*′*	110.61	7.13, brs	114.08	6.93, brs	110.62	7.14, brs
3*′*	148.72		146.46		148.74	
4*′*	149.04		147.92		149.09	
5*′*	111.54	6.98, d (8.1)	111.96	6.94, brs	111.53	6.98, d (8.1)
6*′*	119.27	7.01, d (8.1)	117.71	6.88, d (6.4)	119.34	7.02, d (8.1)
7-OMe			55.90	3.77, s	55.94	3.79, s
3*′*-OMe	55.58	3.77, s			55.58	3.77, s
4*′*-OMe	55.58	3.77, s	55.67	3.77, s	55.61	3.78, s
5-OH		12.14, s		12.10, s		12.11, s
3*′*-OH				9.10, s		

**Table 4:** MIC and MBC of samples to *E*. *coli* ATCC25922, *S. aureus* ATCC6538, and *C. albicans* SC5314.

Compound	*E. coli* ATCC25922	*S. aureus* ATCC6538	*C. albicans* SC5314	*E. coli* ATCC25922	*S. aureus* ATCC6538	*C. albicans* SC5314
	MIC (μM)	MIC (μM)	MIC (μM)	MBC (μM)	MBC (μM)	MBC (μM)
1 1a	25 15	75 75	25 25	>400 >400	>400 >400	>400 >400
2 2a	- -	- -	75 75	- -	- -	>400 >400
3	-	-	75			>400
3a	-	-	50			>400
3b	-	-	25			>400
3c	-	-	75			>400

**Table 5:** Antiproliferative activities (IC _50_) of the compound against MCF-7 human breast cancer cells (mean ± SD, n = 3).

Compound	IC _50_ (μM)
1	100.94 ± 1.83
1a	11.23 ± 0.60
2	125.53 ± 2.76
2a	93.64 ± 1.06
3	>150
3a	10.45 ± 0.45
3b	30.74 ± 0.72
3c	31.00 ± 1.44

## References

[1] Jang D., Jung Y.S., Kim M.S., Oh S.E., Nam T.G., Kim D.O. (2019). Developing and validating a method for separating flavonoid isomers in common buckwheat sprouts using HPLC-PDA. Foods.

[2] Cannas M., Pulina S., Conte P., Del Caro A., Urgeghe P.P., Piga A., Fadda C. (2020). Effect of substitution of rice flour with quinoa flour on the chemical-ohysical, nutritional, volatile and sensory parameters of gluten-free ladyfinger biscuits. Foods.

[3] Irakli M., Lazaridou A., Mylonas I., Biliaderis C.G. (2020). Bioactive components and antioxidant activity distribution in pearling fractions of different greek barley cultivars. Foods.

[4] Siah M., Farzaei M.H., Ashrafi-Kooshk M.R., Adibi H., Arab S.S., Rashidi M.R., Khodarahmi R. (2016). Inhibition of guinea pig aldehyde oxidase activity by different flavonoid compounds: An in vitro study. Bioorg. Chem..

[5] Bao G.L., Zhang Y.L., Yang X.G. (2020). Effect of lemon peel flavonoids on anti-fatigue and anti-oxidation capacities of exhaustive exercise mice. Appl. Biol. Chem..

[6] Lin Z.Y., Lin Y.Y., Shen J.X., Jiang M.J., Hou Y.M. (2020). Flavonoids in *Ageratum conyzoides* L. exert potent antitumor effects on human cervical adenocarcinoma HeLa cells in vitro and in vivo. Biomed. Res. Int..

[7] Qiu J., Jiang Y., Xia L., Xiang H., Feng H., Pu S., Huang N., Yu L., Deng X. (2010). Subinhibitory concentrations of licochalcone A decrease alpha-toxin production in both methicillin-sensitive and methicillin-resistant *Staphylococcus aureus* isolates. Lett. Appl. Microbiol..

[8] Uzel A., Sorkun K., Oncag O., Cogulu D., Gencay O., Salih B. (2005). Chemical compositions and antimicrobial activities of four different Anatolian propolis samples. Microbiol. Res..

[9] Vipin C., Saptami K., Fida F., Mujeeburahiman M., Rao S.E.S., Arun A.B., Rekha P.D. (2020). Potential synergistic activity of quercetin with antibiotics against multidrug-resistant clinical strains of Pseudomonas aeruginosa. PLoS ONE.

[10] Cheng M., Yuan F.Y., Liu J.L., Liu W., Feng J.F., Jin Y., Tu L.X. (2020). Fabrication of Fine Puerarin Nanocrystals by Box-Behnken design to enhance intestinal absorption. Aaps Pharmscitech.

[11] Katsoura M.H., Polydera A.C., Tsironis L., Tselepis A.D., Stamatis H. (2006). Use of ionic liquids as media for the biocatalytic preparation of flavonoid derivatives with antioxidant potency. J. Biotechnol..

[12] Mellou F., Lazari D., Skaltsa H., Tselepis A.D., Kolisis E., Stamatis H. (2005). Biocatalytic preparation of acylated derivatives of flavonoid glycosides enhances their antioxidant and antimicrobial activity. J. Biotechnol..

[13] Lue B.M., Nielsen N.S., Jacobsen C., Hellgren L., Guo Z., Xu X.B. (2010). Antioxidant properties of modified rutin esters by DPPH, reducing power, iron chelation and human low density lipoprotein assays. Food Chem..

[14] Hoang T.K.D., Huynh T.K.C., Nguyen T.D. (2015). Synthesis, characterization, anti-inflammatory and anti-proliferative activity against MCF-7 cells of O-alkyl and O-acyl flavonoid derivatives. Bioorg. Chem..

[15] Araujo K.C.F., Costa E.M.D.B., Pazini F., Valadares M.C., de Oliveira V. (2013). Bioconversion of quercetin and rutin and the cytotoxicity activities of the transformed products. Food Chem. Toxicol..

[16] Copmans D., Orellana-Paucar A.M., Steurs G., Zhang Y., Ny A., Fpubert K., Exarchou V., Siekierska A., Kim Y., De Borggraeve W. (2018). Methylated flavonoids as anti-seizure agents: Naringenin 4′,7-dimethyl ether attenuates epileptic seizures. in zebrafish and mouse models. Neurochem. Int..

[17] Xu P., Hua D., Ma C. (2007). Microbial transformation of propenylbenzenes for natural flavour production. Trends Biotechnol..

[18] Xiao Y., Han F., Lee I.S. (2020). Microbial transformation of licochalcones. Molecules.

[19] Wang X.J., Wang C., Duan L.X., Zhang L.W., Liu H., Xu Y.M., Liu Q.P., Mao T.L., Zhang W., Chen M. (2019). Rational reprogramming of O-methylation regioselectivity for combinatorial biosynthetic tailoring of Benzenediol Lactone Scaffolds. J. Am. Chem. Soc..

[20] Zhong L., Peng L., Fu J., Zou L., Zhao G., Zhao J. (2020). Phytochemical, antibacterial and antioxidant activity evaluation of *Rhodiola crenulata*. Molecules.

[21] Zhu Y.Y., Yao Y., Shi Z.X., Everaert N., Ren G.X. (2018). Synergistic effect of bioactive anticarcinogens from soybean on anti-proliferative activity in MDA-MB-231 and MCF-7 human breast cancer vells in vitro. Molecules.

[22] Liu X.N., Cheng J., Zhu X.X., Zhang G.H., Yang S.C., Guo X.X., Jiang H.F., Ma Y.H. (2020). De Novo biosynthesis of multiple pinocembrin derivatives in *Saccharomyces cerevisiae*. ACS Synth. Biol..

[23] Alseekh S., de Souza L.P., Benina M., Fernie A.R. (2020). The style and substance of plant flavonoid decoration; towards defining both structure and function. Phytochemistry.

[24] Ng K.R., Lyu X.M., Mark R., Chen W.N. (2019). Antimicrobial and antioxidant activities of phenolic metabolites from flavonoid-producing yeast: Potential as natural food preservatives. Food Chem..

[25] Duranoglu D., Uzunoglu D., Mansuroglu B., Arasoglu T., Derman S. (2018). Synthesis of hesperetin-loaded PLGA nanoparticles by two different experimental design methods and biological evaluation of optimized nanoparticles. Nanotechnology.

[26] Wen W., Jin M., Li K., Liu H., Xiao Y., Zhao M., Alseekh S., Li W., de Abreu E.L.F., Brotman Y. (2018). An integrated multi-layered analysis of the metabolic networks of different tissues uncovers key genetic components of primary metabolism in maize. Plant J..

[27] Magozwi D.K., Dinala M., Mokwana N., Siwe-Noundou X., Krause R.W.M., Sonopo M., McGaw L.J., Augustyn W.A., Tembu V.J. (2021). Flavonoids from the genus Euphorbia: Isolation, structure, pharmacological activities and structure-activity relationships. Pharmaceuticals.

[28] Kong W.J., Zhao Y.L., Xing X.Y., Ma X.P., Sun X.J., Yang M.H., Xiao X.H. (2015). Antibacterial evaluation of flavonoid compounds against E-coli by microcalorimetry and chemometrics. Appl. Microbiol. Biot..

[29] Liang F., Zhang H., Gao H., Cheng D., Zhang N., Du J., Yue J.M., Du P., Zhao B.B., Yin L. (2021). Liquiritigenin decreases tumorigenesis by inhibiting DNMT activity and increasing BRCA1 transcriptional activity in triple-negative breast cancer. Exp. Biol. Med..

[30] Pateliya B., Burade V., Goswami S. (2021). Enhanced antitumor activity of doxorubicin by naringenin and metformin in breast carcinoma: An experimental study. N S Arch. Pharmacol..

[31] Korga-Plewko A., Michalczyk M., Adamczuk G., Humeniuk E., Ostrowska-Lesko M., Jozefczyk A., Iwan M., Wojcik M., Dudka J. (2020). Apigenin and hesperidin downregulate DNA repair genes in MCF-7 breast cancer cells and augment doxorubicin toxicity. Molecules.

[32] Ajji P.K., Walder K., Puri M. (2020). Combination of balsamin and flavonoids induce apoptotic effects in liver and breast cancer cells. Front. Pharmacol..

[33] Rahideh S.T., Shidfar F., Nourbakhsh M., Hoseini M., Koohdani F., Entezam M., Keramatipour M. (2016). The individual or combinational effects of Hesperetin and Letrozole on the activity and expression of aromatase in MCF-7 cells. Cell Mol. Biol..

[34] Wen L.R., Jiang Y.M., Yang J.L., Zhao Y.P., Tian M.M., Yang B. (2017). Structure, bioactivity, and synthesis of methylated flavonoids. Ann. N. Y. Acad. Sci..

[35] Katayama K., Masuyama K., Yoshioka S., Hasegawa H., Mitsuhashi J., Sugimoto Y. (2007). Flavonoids inhibit breast cancer resistance protein-mediated drug resistance: Transporter specificity and structure-activity relationship. Cancer Chemother. Pharmacol..

[36] Fan X., Bai J., Zhao S., Hu M., Sun Y., Wang B., Ji M., Jin J., Wang X., Hu J. (2019). Evaluation of inhibitory effects of flavonoids on breast cancer resistance protein (BCRP): From library screening to biological evaluation to structure-activity relationship. Toxicol. Vitro.

[37] Brusselmans K., Bono F., Collen D., Herbert J.M., Carmeliet P., Dewerchin M. (2005). A novel role for vascular endothelial growth factor as an autocrine survival factor for embryonic stem cells during hypoxia. J. Biol. Chem..

[38] Kitagawa S., Nabekura T., Takahashi T., Nakamura Y., Sakamoto H., Tano H., Hirai M., Tsukahara G. (2005). Structure-activity relationships of the inhibitory effects of flavonoids on P-glycoprotein-mediated transport in KB-C2 cells. Biol. Pharm. Bull..

[39] Plochmann K., Korte G., Koutsilieri E., Richling E., Riederer P., Rethwilm A., Schreier P., Scheller C. (2007). Structure-activity relationships of flavonoid-induced cytotoxicity on human leukemia cells. Arch. Biochem. Biophys..

